# Testing the ability for autonomous oral hygiene in hospitalized geriatric patients—clinical validation study

**DOI:** 10.1007/s00784-020-03402-5

**Published:** 2020-06-23

**Authors:** Ina Manuela Schüler, Barbara Kurtz, Roswitha Heinrich-Weltzien, Thomas Lehmann, Anja Kwetkat

**Affiliations:** 1 Section of Preventive and Pediatric Dentistry, Department of Orthodontics, Jena University Hospital, Bachstraße 18, D -, 07743 Jena, Germany; 2 Institute of Medical Statistics, Computer Sciences and Documentation, Jena University Hospital, Jena, Germany; 3 Department of Geriatric Medicine, Jena University Hospital, Jena, Germany

**Keywords:** Oral hygiene, Geriatric assessment, Self-management, Timed Test for Money Counting

## Abstract

**Objectives:**

This study aimed to evaluate if the Timed Test for Money Counting (TTMC) complemented with testing the range of shoulder motion by griping the backside of the neck (NG) predicts the ability of geriatric inpatients to perform effective plaque reduction by autonomously conducted oral hygiene.

**Material and methods:**

This clinical validation study involved 74 hospitalized geriatric inpatients, 48 (64.9%) females, aged between 66 and 98 years (mean age 84.1 years). Oral health status was examined. Dental plaque was assessed with the Turesky modified Quigley-Hein Index (TI) on teeth and the Denture Hygiene Index (DHI) on removable dentures. The performance and duration of TTMC and NG were recorded. After autonomous tooth brushing and denture cleaning by the patient, dental plaque was scored again with the TI and DHI. Geriatric assessment data were collected from medical records.

**Results:**

Forty-nine (66.2%) geriatric inpatients completed the TTMC&NG successfully. Passing the TTMC&NG was significantly associated with better plaque removal on teeth and dentures by autonomously conducted oral hygiene. The sensitivity of the TTMC&NG for above average plaque reduction was 86.4% on teeth and 77.8% on dentures. The test revealed a negative predictive value of 75.0% to detect below average plaque reduction on teeth and 72.7% on dentures.

**Conclusions:**

The TTMC&NG served as a suitable predictor for the ability of geriatric inpatients to perform autonomously effective tooth brushing and denture cleaning.

**Clinical relevance:**

This simple and short test might help the medical staff to identify geriatric inpatients unable to perform effective oral hygiene by themselves.

## Introduction

Hospitalized geriatric patients bear a double burden—besides the functional and cognitive limitations accompanying the aging process they have to cope with multimorbidity and acute medical conditions. In this situation, efforts should be undertaken to avoid additional concomitant oral health-related problems due to insufficient oral hygiene.

Maintaining good oral health is important for geriatric patients because it is strongly related to nutrition intake, decreased risk of respiratory and cardiovascular diseases, and better quality of life [1–4]. Furthermore, chronic periodontal inflammations persisting over many years are assumed to promote the inflammaging process, increasing the susceptibility for infections, malignancies, and autoimmune diseases [5]. Nevertheless, studies report high prevalence rates of dental caries, periodontal disease, tooth loss, and mucositis [6–8]. Gerontologists are concerned that poor oral health will become a new geriatric syndrome [9].

Oral cleanliness is crucial for maintaining oral health. Neglect of oral hygiene threatens the patients’ well-being, and general health might become jeopardized [10]. Plaque-induced oral diseases were identified as a major risk to a patient’s ability to eat, communicate, and socialize [11]. Furthermore, dental plaque might influence the course of respiratory infections [2, 12, 13]. There is good evidence that effective plaque removal reduces progression or occurrence of respiratory diseases among frail elderly nursing home residents, hospitalized patients, and especially intensive care patients [2, 14–16].

Regarding frail and medically compromised geriatric patients, the question arises which patients are still capable to perform sufficient oral hygiene by themselves and which patients need assistance or oral hygiene to be overtaken by nursing staff. This decision is reported to be difficult for dentists [17] and probably even more challenging for geriatric medical and nursing staff. A short and easy-to-use test for detecting geriatric patients with impaired ability to perform effective oral hygiene by non-dental professionals might help in this decision process.

Tooth brushing and denture cleaning require a certain level of manual dexterity, visual acuity, procedural and cognitive skills, and sufficient mobility of the shoulder and elbow joints. The Timed Test for Money Counting (TTMC) is a validated and established geriatric assessment tool evaluating manual skills, visual acuity, and cognitive capacity [18]. It is simple to perform and requires about 5 min. The TTMC was successfully validated to evaluate geriatric patients’ ability to manage insulin therapy autonomously [19], which requires similar skills as oral hygiene. Except the shoulder mobility, all general skills necessary to perform oral hygiene might be assessed with the TTMC. The range of shoulder mobility can be tested short and easily inviting the patient to grip the backside of the neck (NG). The combination of both tests—TTMC&NG—covers the main skills necessary to perform tooth brushing and denture cleaning.

The aim of this study was to evaluate if the TTMC&NG could serve as a suitable tool for testing the ability of geriatric inpatients to perform effective plaque reduction by autonomous tooth brushing and denture cleaning.

## Material and methods

This clinical validation study was performed between May 2016 and May 2017 at the Geriatric Clinic of Jena University Hospital.

### Study sample

All patients hospitalized during the study period were eligible to enrollment. The mean age was used to divide the study sample in one younger (under mean) and one older (over mean) age group.

The sample size was estimated to the number of patients necessary to evidence a strong association between passing or failing the TTMC&NG and plaque reduction rates on teeth and dentures with an effect size of *d* ≥ 0.5, 90% power, and alpha = 0.05. Forty-two patients with natural teeth and 42 with removable dentures had to be included.

### Oral examination

All patients were examined in the patient rooms of the geriatric ward by one calibrated dentist (B.K.) using a ball-end probe and an illuminated mirror (DenLite®, Miltex, USA) after drying the teeth with cotton rolls and without use of radiographs. For examination, patients were sitting on a chair or lying in bed in supine position.

Dental caries was scored in accordance with the WHO criteria for caries diagnostics [20] using the DMFT index.

Gingival health was evaluated by the periodontal screening index (PSI) [21]. The gingiva surrounding all teeth was examined with a standardized ball-end probe with a black band from 3.5 to 5.5 mm using light probing force. After dividing the dentition into sextants, the highest score of each sextant was recorded.

Dental plaque was scored in all patients with natural tooth crowns by Turesky modified Quigley-Hein Index (TI) [22] after staining all teeth surfaces with Rondells Blue© (Directa, Sweden) before (t0) and after autonomously tooth brushing (t1). Tooth brushing was performed in the washing facilities of the patients’ rooms. Plaque on dentures was scored in all patients with removable dentures by Denture Hygiene Index (DHI) [23] by visual inspection without staining before (t0) and after (t1) denture cleaning performed by the inpatient.

Prior to the examinations, the dentist (B.K.) was trained by an experienced dentist and epidemiologist (I.M.S.). The intra- und inter-examiner reproducibility was assessed by the weighted kappa (*k*_w_) statistics. The *k*_w_ values for inter-examiner reproducibility ranged from 0.89 for the TI Index to 0.91 for the DHI. Intra-examiner reproducibility ranged from 0.94 to 0.97 (examiner B.K.) and 0.93 to 0.97 (examiner I.M.S.) for both indices. The inter- and intra-examiner reproducibility for the TTMC&NG was 1.0 for both examiners.

### Timed Test for Money Counting

The Timed Test for Money Counting (TTMC) measures the time a person needs to open a standardized purse, to take out the standardized amount of money in a standardized denomination, and to count it with the aim to identify persons in need for increased health care [18].

In the present study, a conventional purse (about 12 × 9cm) with one compartment for bills and one with push button for coins contained 9.80€ in the following denominations: 1 × 5€ note in the compartment for bills and 1 × 2 € coin, 2 × 1 € coins, 1 × 50 cent coin, and 3 × 10 cent coins in the compartment for coins.

The purse was shown to the patient; it was closed again and handed over to the patient, with the request to count the money in the purse. The time was measured in seconds with a stopwatch. If the result was correct, the time was stopped and noted. If an incorrect result was reported, the patient was notified that the finding was incorrect and she/he was allowed to try again. The time measurement continued. The test was scored as failed after three failed attempts, when more than 300 s (5 min) were needed or if the patient canceled the test due to lack of self-perceived ability or visual or motoric problems. The TTMC uses cutoff times of less than 45 s to identify independent patients, 45–70 s for patients at risk for neediness, and more than 70 s for patients at risk for extensive assistance [18, 24]. Additionally, difficulties related to fine and gross motor skills and visual and cognitive limitations during test performance were recorded.

### Range of movement for shoulder motion

Restricted range of shoulder motion was detected by asking the patients to grip the backside of their neck (NG) while standing or sitting in an upright position without props. The examiner decided if the shoulder range of movement was sufficient to reach the backside of the neck or not. It was precluded that patients who are able to reach their own backside of the neck with both hands or at least with the dominant hand are also able to reach their oral cavities for tooth brushing. Complaint of pain was recorded.

The combination of TTMC&NG was scored as failed, if one of the tests were not completed successfully.

### Oral health self-assessment questionnaire

A simple paper-based questionnaire with mainly closed questions was used to record the patient’s self-perceived oral health, oral hygiene habits, and independency of oral hygiene at home and at the hospital. Furthermore, patients were asked if they perceive having problems in performing oral hygiene by themselves. Wording of the questions was concise, unambiguous, and asked in a personal interview.

### Collection of geriatric assessment data

Comprehensive geriatric assessment is part of the daily routine by geriatric physicians or medical staff and conducted with validated and standardized tools. It is a multidimensional interdisciplinary diagnostic process focused on determining patients’ medical, psychological, and functional capability aiming to develop a coordinated and integrated plan for treatment and long-term follow-ups [25]. In this study, the results of standard geriatric assessment tests (Barthel Index [26], Geriatric Depression Scale by Yesavage [27], and Mini Mental State Examination by Folstein [28]) conducted in daily routine by geriatric staff were collected from the medical records of the patients.

The Barthel Index has been developed to assess basic everyday functions in patients with neurological or musculoskeletal impairment [26]. The objective is to determine the degree of independence concerning the two main areas of self-sufficiency: personal care (food intake, personal hygiene, toilet use) and mobility (locomotion, climbing stairs). Higher score indicates greater independence of the patient but only for the activities observed in the test. A score of 100 points characterized an independent patient, 95–85 points a patient requiring low-level care, 80–35 points a patient needing medium-level care, and 30–0 points a patient demanding high-level care [29].

Cognitive function was assessed using the German version of the Mini Mental State Examination (MMSE) [28], testing cognitive aspects of mental function, e.g., orientation, registration, attention, memory, and ability to follow verbal and written commands. The MMSE has a score ranging between 0 and 30. The cognitive impairment is assumed severe with a score ≤ 17 points and as mild with a score of 18–23 points. Patients achieving scores of 24–30 points were considered without significant cognitive impairment [30].

The German version of the Geriatric Depression Scale after Yesavage (GDS) [27] was used to detect depressed mood and signs of depressive disorders. It consists of 15 questions, and each answer indicating depressive mood is counted. The score ranges from 0 to 15. A score of 0–4 is considered to be within the normal range, 5–9 indicates mild depression, and score 10 or more indicates moderate to severe depression [31].

### Data analysis and statistics

Data collection was performed with Microsoft Excel® 2013 (Microsoft Corp., Redmond, USA) and statistical analysis by SPSS for Windows (IBM SPSS Statistics for Windows, Version 20.0. Armonk, NY: IBM Corp). Mean values were analyzed by *t* test and ANOVA and categorical data by chi-square test. Spearman’s rho was calculated to detect correlation between continuous variables.

For test validation, 2 × 2 tables were used to assign true positive (TP), true negative (TN), false positive (FP), and false negative (FN) test results and to calculate sensitivity (SE), specificity (SP), positive predictive value (PPV), and negative predictive value (NPV) by following formulas: SE = TP/(TP + FN) × 100, SP = TN/(TN + FP) × 100), PPV = TP/(TP + FP) × 100, and NPV = TN/(FN + TN) × 100.

Significance level was set at *p* ≤ 0.05.

## Results

From 809 eligible patients, 74 (9.2%) hospitalized geriatric inpatients aged between 66 and 98 years with mean age of 84.1 (SD = 5.8) years were included, 64.9% were females. Causes for nonparticipation were severe medical conditions, quarantine due to infections or colonization with multi-resistant pathogens, or lack of willingness. In the study population, 46 patients had natural teeth; two of them had only tooth roots without crowns impeding the assessment of TI. Thirty-seven of the dentate patients and all 28 toothless patients wore removable dentures. Due to the clinically frequently met situation of dentate patients wearing partial dentures, the number of patients with removable dentures (*n* = 63) exceeded the required sample size.

### Oral health

Acute dental treatment need was diagnosed in 60.8% of the patients, while acute oral pain was reported by 9.5% (Table 1).

**Table 1:** Oral health parameters in geriatric patients

	All	Age ≤ 84 years	Age > 84 years	Males	Females
Study population % (*n*) [95% CI]	100.0 (74)	52.7 (39) [41.9–63.5]	47.3 (35) [36.5–58.1]	35.1 (26) [24.3–45.9]	64.9 (48) [54.1–75.7]
Dental status					
Dentate % (*n*) [95% CI]	62.2 (46) [50.0–73.0]	71.8 (28) [56.8–85.0]	51.4 (18) [34.5–69.2]	65.4 (17) [47.4–83.3]	60.4 (29) [46.3–73.3]
Number of teeth MW (SD)	6.9 (0.0)	8.4 (8.8)	5.3 (6.8)	6.4 (7.2)	7.3 (8.5)
DMFT MW (SD)	26.3 (3.1)	26.2 (3.4)	26.5 (2.7)	26.4 (2.4)	26.3 (3.4)
Dentures % (*n*) [95% CI]	85.1 (63) [75.7–93.2]	79.5 (31) [66.7–91.4]	91.4 (32) [81.8–100.0]	84.6 (22) [70.8–96.0]	85.4 (41) [74.1–95.0]
Acute oral pain % (*n*) [95% CI]	9.5 (7) [2.7–16.2]	10.3 (4) [2.2–20.0]	8.6 (3) [0.0–18.9]	15.4 (4) [3.6–32.0]	6.3 (3) [0.0–14.3]
Acute dental treatment need % (*n*) [95% CI]	60.8 (45) [50.0–71.6]	69.2 (27) [55.3–83.3]	51.4 (18) [34.4–67.7]	57.7 (15) [38.1–76.7]	62.5 (30) [48.0–75.5]
Periodontal status					
PSI = 0% (*n*) [95% CI]	10.9 (5) [2.2–21.7]	17.9 (5) [4.2–33.3]	0.0 (0) [0.0–0.0]	5.9 (1) [0.0–18.8]	13.8 (4) [3.1–26.7]
PSI = 1–2% (*n*) [95% CI]	45.7 (21) [32.6–58.7]	39.3 (11) [21.2–58.6]	55.6 (10) [31.6–81.2]	58.8 (10) [33.3–84.2]	37.9 (11) [20.8–57.7]
PSI = 3–4% (*n*) [95% CI]	43.5 (20) [28.3–58.7]	42.9 (12) [23.1–62.1]	44.4 (8) [18.8–68.4]	35.3 (6) [13.4–60.0]	48.3 (14) [30.8–66.7]
Dental plaque					
TI at t0 MW (SD)	2.74 (1.07)	2.61 (1.12)	2.94 (1.00)	3.11 (1.17)	2.53 (0.96)
DHI at t0 MW (SD)	0.45 (0.31)	0.44 (0.32)	0.47 (0.3)	0.53 (0.36)	0.41 (0.28)
Self-evaluation					
My oral hygiene is very good % (*n*) [95% CI]	5.4 (4) [1.4–10.8]	5.1 (2) [0.0–13.0]	5.7 (2) [0.0–14.3]	7.7 (2) [0.0–19.0]	4.2 (2) [0.0–10.6]
My oral hygiene is good % (*n*) [95% CI]	68.9 (51) [58.1–78.4]	69.2 (27) [53.1–83.3]	68.6 (24) [53.1–84.2]	61.5 (16) [40.6–80.6]	72.9 (35) [60.0–84.8]
My oral hygiene is satisfactory % (*n*) [95% CI]	25.7 (19) [16.2–36.5]	25.6 (10) [13.3–41.2]	25.7 (9) [12.1–40.6]	30.8 (8) [13.3–50.0]	22.9 (11) [11.8–35.6]
I have problems in performing oral hygiene % (*n*) [95% CI]	16.2 (12) [8.1–25.7]	17.9 (7) [7.0–31.6]	14.3 (5) [4.2–27.6]	26.9 (7) [10.3–43.8]	10.4 (5) [2.3–20.0]
I perform oral hygiene without help % (*n*) [95% CI]	87.8 (65) [79.7–94.6]	82.1 (32) [6.5–31.2]	94.3 (33) [84.8–100.0]	84.6 (22) [69.0–96.7]	89.6 (43) [80.8–97.7]

Chi-square test for categorical data. ANOVA test for mean values. No significances between age groups and sex

All results concerning oral health are shown in Table 1. Within the study population, 62.2% were dentate with a mean number of 6.9 remaining teeth. More patients aged over 84 years were edentulous compared with those up to 84 years. The number of teeth was higher in patients up to 84 years and in females. Caries experience revealed no significant age- or sex-related differences but in means 1.7 teeth with untreated carious lesions.

Prevalence of dentures was 85.1%. Complete dentures were more frequently in the upper (54.1%) than in the lower jaw (41.9%).

Gingivitis and periodontitis increased by age and gingivitis occurred more often in males and periodontitis in females, both without statistical significance. Slightly more dental plaque was recorded in older patients compared with the younger age group and in males compared with females.

The majority of patients evaluated their oral hygiene as good, females slightly more frequently than males. Problems in performing oral hygiene were reported by 16.2% of the patients, more often by males compared with females. The majority of patients performed oral hygiene at home or in the hospital without any help.

### Geriatric assessment

Table 2 shows the results of the geriatric assessment.

**Table 2:** Parameters of geriatric assessment tests in geriatric patients

	All	Age ≤ 84 years	Age > 84 years	Males	Females
TTMC					
TTMC passed % (*n*) [95% CI]	70.3 (52) [59.5–81.0]	61.5 (24) [45.2–76.2]	80.0 (28) [65.4–91.7]	69.2 (18) [50.0–88.0]	70.8 (34) [57.1–83.3]
TTMC&NG passed % (*n*) [95% CI]	66.2 (49) [55.4–75.7]	61.5 (24) [45.5–76.9]	71.4 (25) [65.3–86.2]	69.*2* (18) [50.0–88.0]	64.6 (31) [50.0–78.3]
Barthel Index					
Low-level care % (*n*) [95% CI]	23.0 (17) [13.5–33.8]	25.6 (10) [12.8–40.0]	20.0 (7) [8.3–35.3]	19.2 (5) [4.3–35.7]	25.0 (12) [13.2–37.0]
Medium-level care % (*n*) [95% CI]	68.9 (51) [58.1–79.7]	69.2 (27) [53.9–83.8]	68.6 (24) [52.4–82.5]	69.2 (18) [50.0–88.0]	68.8 (33) [56.1–81.6]
High-level care % (*n*) [95% CI]	8.1 (6) [2.7–14.9]	5.1 (2) [0.0–13.5]	11.4 (4) [2.6–23.3]	11.5 (3) [0.0–24.0]	6.3 (3) [0.0–14.0]
MMSE					
No significant cognitive impairment % (*n*) [95% CI]	68.9 (51) [58.1–79.7]	64.1 (25) [48.5–78.9]	74.3 (26) [60.0–88.6]	69.2 (18) [50.0–86.9]	68.8 (33) [54.3–81.8]
Slight/moderate cognitive impairment % (*n*) [95% CI]	18.9 (14) [10.8–29.7]	25.6 (10) [12.5–41.2]	11.4 (4) [2.8–23.1]	15.4 (4) [30.5–30.4]	20.8 (10) [10.0–34.6]
Severe cognitive impairment % (*n*) [95% CI]	12.2 (9) [5.4–20.2]	10.3 (4) [2.3–20.5]	14.3 (5) [3.1–25.8]	15.4 (4) [3.0–30.8]	10.4 (5) [2.2–19.5]
Geriatric Depression Scale (GDS)					
No depression % (*n*) [95% CI]	64.9 (48) [54.1–75.7]	64.1 (25) [47.5–79.1]	65.7 (23) [48.8–81.6]	57.7 (15) [37.9–75.8]	68.8 (33) [55.6–81.3]
Slight depression % (*n*) [95% CI]	27.0 (20) [17.6–36.5]	25.6 (10) [11.8–40.9]	28.6 (10) [14.3–44.1]	38.5 (10) [21.7–57.1]	20.8 (10) [8.7–32.0]
Moderate/major depression % (*n*) [95% CI]	8.1 (6) [2.7–14.9]	10.3 (4) [2.4–21.2]	5.7 (2) [0.0–15.1]	3.8 (1) [0.0–12.5]	10.4 (5) [2.4–20.4]

*Chi-square test, no significances between age groups and sex

The TTMC was completed successfully by 70.3% (*n* = 52) of the geriatric inpatients. The participants aged over 84 years passed the test more frequently than the younger ones. They needed between 21 and 347 s (mean, 90.7; SD = 68.7 s) to complete the test. The duration did not correlate by age (*r* = 0.07; *p* = 0.553). Patients passing the TTMC needed significantly less time (67.2.; SD = 42.6 s) compared with those who failed (146.1; SD = 86.0 s; *p* = 0.000). Three patients who passed the TTMC failed the NG. All age- or sex-related differences regarding the geriatric assessment results were without statistical significance.

Performance in activities of daily living and mobility assessed with the Barthel Index revealed that 77.0% (*n* = 57) of the geriatric inpatients required medium- or high-level care. The need for high-level care was more prevalent in patients aged over 84 years and in males yet without statistical significance.

One third of the patients showed cognitive impairment assessed with the MMSE. Severe cognitive impairment was slightly more prevalent in patients aged over 84 years and in males.

Signs of depression were detected in one third of the geriatric patients by the GDS. Slight depression was more prevalent in males and moderate to major depression in females but without statistical significance.

### Plaque reduction

Table 3 demonstrates the factors influencing plaque reduction on teeth and dentures in the participating geriatric inpatients. Performing tooth brushing and denture cleaning autonomously, patients reached reductions of mean TI (TI diff t0–t1) between 0.0 and 1.8 (mean 0.8; SD = 0.5) and of mean DHI (DHI diff t0–t1) between 0.0 and 0.8 (mean 0.2; SD = 0.2). Female patients reached higher levels of plaque reduction on teeth compared with males.

**Table 3:** Factors influencing plaque reduction on teeth (measured by TI) and on dentures (measured by DHI) in geriatric inpatients

Factor		TI diff t0–t1 mean (SD)	p*	DHI diff t0–t1 mean (SD)	p*
Study population		0.8 (0.5)		0.2 (0.2)	
Age	< 84 years	0.8 (0.2)	*0.319*	0.2 (0.2)	*0.115*
	≥ 85 years	0.9 (0.2)		0.3 (0.2)	
Sex	Males	0.9 (0.4)	*0.393*	0.2 (0.2)	*0.191*
	Females	0.3 (0.2)		0.3 (0.2)	
Oral hygiene (self-evaluation)	Very good	0.3 (0.3)	*0.129*	0.3 (0.3)	*0.485*
	Good	0.9 (0.4)		0.3 (0.2)	
	Satisfactory	0.8 (0.5)		0.2 (0.2)	
Subjective problems in performing oral hygiene	Yes	1.1 (0.4)	*0.111*	0.3 (0.2)	*0.192*
	No	0.8 (0.5)		0.2 (0.2)	
Performing oral hygiene without help	Yes	0.8 (0.5)	*0.682*	0.2 (0.2)	*0.432*
	No	0.9 (0.4)		0.3 (0.2)	
TTMC time cutoffs	< 45 s	0.9 (0.6)	***0.003***	0.3 (0.2)	*0.081*
	45–70 s	1.0 (0.4)		0.3 (0.2)	
	> 70 s	0.5 (0.3)		0.2 (0.1)	
TTMC	Passed	0.9 (0.5)	***0.016***	0.3 (0.2)	***0.031***
	Failed	0.6 (0.3)		0.2 (0.1)	
TTMC&NG	Passed	0.9 (0.4)	***0.015***	0.3 (0.2)	***0.014***
	Failed	0.5 (0.3)		0.2 (0.1)	
Barthel Scale	Low-level care	0.8 (0.5)	*0.887*	0.3 (0.2)	*0.412*
	Medium-level care	0.8 (0.4)		0.2 (0.2)	
	High-level care	0.7 (0.5)		0.2 (0.1)	
MMSE	No significant cognitive impairment	0.8 (0.4)	*0.300*	0.3 (0.2)	*0.550*
	Slight/moderate cognitive impairment	1.0 (0.6)		0.3 (0.2)	
	Severe cognitive impairment	0.7 (0.3)		0.2 (0.2)	
Geriatric Depression Scale (GDS)	No depression	0.8 (0.5)	*0.653*	0.3 (0.2)	*0.089*
	Slight depression	0.8 (0.3)		0.2 (0.1)	
	Moderate/major depression	1.0 (0.7)		0.4 (0.2)	

*ANOVA test. Significant *p*-values are displayed in bold

The self-evaluation of oral hygiene did not coincide with the plaque reduction rates. Patients performing oral hygiene without help reached very slightly higher plaque reduction compared with those receiving help.

Plaque reduction was not significantly influenced by depression, assessed by GDS; level of care, evaluated by Barthel Scale; or cognitive impairment, measured by MMSE.

Passing the TTMC as well as the TTMC&NG was significantly associated with better plaque removal on teeth and dentures (Table 3).

There was observed a weak correlation between the time needed to complete the test and the plaque reduction on teeth (*r* = − 0.3, *p* = 0.028, Spearman’s correlation test) and dentures (*r* = − 0.2, *p* = 0.090, Spearman’s correlation test). However, using the cutoffs of the TTMC, patients needing more than 70 s to complete the test had lower plaque reduction rates on teeth (*p* = 0.003; ANOVA) and dentures (*p* = 0.081; ANOVA) (Table 3).

The occurrence of problems during the performance of TTMC was significantly associated with less plaque removal only for cognitive and visual problems (Table 4). In patients with cognitive problems during TTMC, significantly lower plaque reduction was observed for tooth brushing and denture cleaning. Additionally, visual problems were associated with significantly lower plaque removal on dentures. All other problems did not lead to significant lower plaque reduction rates (Table 4).

**Table 4:** Problems during TTMC&NG influencing plaque reduction on teeth (measured by TI) and on dentures (measured by DHI) in geriatric patients

Problems occurring during TTMC&NG		*n*	TI diff t0–t1 mean (SD)	p*	*n*	DHI diff t0–t1 mean (SD)	p*
Any problems during TTMC	Yes	32	0.9 (0.3)	0.280	44	0.2 (0.2)	0.435
	No	12	0.8 (0.5)		16	0.3 (0.2)	
Fine motor skill problems	Yes	22	0.8 (0.5)	0.519	29	0.3 (0.2)	0.774
	No	22	0.9 (0.4)		31	0.2 (0.2)	
Gross motor skill problems	Yes	16	0.8 (0.5)	0.621	21	0.3 (0.2)	0.183
	No	28	0.8 (0.4)		39	0.2 (0.2)	
Cognitive problems	Yes	12	0.5 (0.3)	**0.007**	22	0.2 (0.1)	**0.009**
	No	32	0.9 (0.4)		38	0.3 (0.2)	
Visual problems	Yes	14	0.7 (0.5)	0.167	22	0.2 (0.1)	**0.017**
	No	30	0.9 (0.4)		38	0.3 (0.2)	
Painful shoulder motion	Yes	17	0.8 (0.5)	0.946	24	0.2 (0.2)	0.719
	No	27	0.8 (0.5)		36	0.3 (0.2)	

*ANOVA test. Significant values are displayed in bold

### Validation

The mean difference of the plaque index between t0 and t1 was used to group the plaque reduction rates in above and below average. Passing the TTMC&NG was significantly associated with above average plaque reduction on teeth (*p* = 0.044, chi-square test) and dentures (*p* = 0.033, chi-square test). 2 × 2 tables (Table 5) were used to calculate SE, SP, NPV, and PPV of the TTMC &NG and its time cutoffs for above and below average plaque reduction. The TTMC&NG had a higher sensitivity for above average plaque removal on teeth (86.4%) than on dentures (77.8%). Failing the test predicts with high probability below average tooth and denture plaque reduction (NPV 75.0% and 72.7%, respectively). The time cutoff of the TTMC failed to produce higher test results compared with the TTMC&NG, except the NPV for plaque reduction on dentures (84.4% vs. 72.7%, respectively).

**Table 5:** 2 × 2 tables contrasting the passing/failure of the TTMC&NG with above and below average plaque reduction rates in geriatric patients

	TTMC&NG passed *n* (%) [positive outcome]	TTMC&NG failed *n* (%) [negative outcome]	< 70 s *n* (%) [positive outcome]	> 70 s n (%) [negative outcome]
Plaque reduction on teeth				
Above average* [positive outcome]	19 (43.2)	3 (6.8)	19 (43.2)	3 (6.8)
Below average* [negative outcome]	13 (29.5)	9 (20.5)	11 (25.0)	11 (25.0)
Plaque reduction on dentures				
Above average# [positive outcome]	21 (33.3)	6 (9.5)	14 (23.3)	9 (15.0)
Below average# [negative outcome]	20 (31.7)	16 (25.4)	18 (30.0)	19 (31.6)

*Mean TI diff t0–t1 = 0.84; #mean DHI diff t0–t1 = 0.24

## Discussion

To the author’s knowledge, this is the first study evaluating the predictability of effective tooth brushing and denture cleaning by using an established geriatric assessment tool—the TTMC—complemented with testing the range of shoulder mobility by simply griping the backside of the neck.

The TTMC&NG seems to be a suitable tool to predict the ability of geriatric inpatients to perform autonomously effective oral hygiene. The high sensitivity shows that the large majority of participants reaching above average plaque reduction also passed the TTMC&NG. Furthermore, the high NPV demonstrated that participants failing the test reached only below average plaque reductions with high probability. Therefore, the TTMC&NG had a better potential to detect geriatric inpatients with deficiencies in performing effective oral hygiene independently compared with the 70 s time cutoff of the TTMC.

Substantial advantages of the TTMC&NG were the simple and short procedure [18, 19]. The expenditure of time is only about 5 min, and the test might be performed within the geriatric assessment, as all geriatric inpatients pass comprehensive geriatric assessment at admission to the geriatric department. Good reliability and validity of the TTMC was already reported [18]. The TTMC requires practical skills relevant to activities of daily living and specifically to oral hygiene: gross and fine motor skills of hand and fingers, visual skills, and cognitive and executive function [19]. Additionally, sufficient mobility of the shoulder is necessary to raise a toothbrush into the mouth and to execute brushing movements. Therefore, the TTMC was complemented with a test for range of shoulder mobility. This test included as a short and simple exercise the griping of the own backside of the neck. The expenditure of time is less than 1 min, and impairment of shoulder motility can be easily detected.

Dentists reported difficulties in estimating the ability on cognitively impaired older patients to perform oral hygiene and desired a reliable and easy-to-use assessment tool [17]. The “Dental Activities Test (DAT)” [32] was proposed as a reliable and valid clinical tool for measuring dentally related function in cognitively impaired older adults. The DAT was moderately associated with dental plaque on teeth and not with plaque on dentures [32]. The correlation between the DAT scores and the ability of plaque reduction is not reported. Furthermore, this tool was designed for use in dental environments and requires dentally trained staff for conducting and interpreting the test. In contrast to the DAT, the proposed TTMC&NG does not require dental environment or staff and is therefore suitable to be performed in standard medical settings like geriatric clinics, hospitals, nursing homes, or medical doctor’s surgeries.

The self-perceived quality of oral hygiene, reported by geriatric inpatients, did not correlate with the actual ability to perform plaque reduction. The standard geriatric assessment tests such as MMSE, GDS, and Barthel Score were not able to detect geriatric inpatients with deficiencies in performing oral hygiene. The Resident Assessment Instrument (RAI), a comprehensive, valid, and reliable tool to assess geriatric residents’ clinical and functional status to monitor and improve quality of care, might reveal information to evaluate geriatric resident’s competence to perform autonomously effective tooth and denture cleaning. This shall be subject of further study although the RAI assesses oral health-related issues with low reliability [33] and validity [34, 35] underestimating oral problems and lacking to record dental plaque.

The amount of time needed to perform the TTMC played a minor role to detect deficient oral hygiene in geriatric inpatients. More important than the time needed to perform the TTMC were the consideration of problems occurring during test performance. Except the painful shoulder motion, all observed problems were associated with higher frequency to fail the TTMC&NG. It seems that patients did not decline oral hygiene due to pain experience in the shoulder. Missing or limited shoulder mobility instead hindered tooth brushing. Furthermore, cognitive and visual impairment were associated with lower plaque reduction rates. Inconsistently, patients with lower MMSE did not show lower plaque reduction rates. It is assumed that the MMSE has low sensitivity to detect patients with cognitive impairment affecting the performance of oral hygiene. This observation is in line with a recent systematic review reporting about the inconsistent findings regarding the interaction between cognitive impairment and oral health [36, 37].

Estimating the ability of performing oral hygiene with the TTMC&NG should consider individual coping skills of the patients. Geriatric inpatients might have developed routines or behaviors compensating impaired functions. For example, in this study, two female patients with very low visual skills performed the TTMC quickly and confidently, because both were working their whole life as sales clerk and counting money was a more familiar exercise than tooth brushing. In these cases, the predictability of the TTMC regarding the ability for oral hygiene is less reliable.

Compared with representative older German people aged between 75 and 100 years in need of care [8], geriatric inpatients had slightly higher caries experience (26.3 vs. 24.5), lower prevalence of edentulism (37.8% vs. 53.7%), and lower number of natural teeth (6.9 vs. 5.6) [8]. Normal ability to perform oral hygiene was observed in 22.5% of 75–100-year-old persons in need for care [8], which is in contrast to our finding of 70.2% patients passing the TTMC&NG. In that representative study, the ability to perform oral hygiene was evaluated by dentists involved in the oral examination of the patients. The evaluation was based solely on the dentist’s professional perception, without use of any competence test. If dentists acknowledged difficulties in estimating the ability of impaired older patients to perform oral hygiene [17], it can be assumed that non-dental professionals like geriatric medical staff encounter comparable or even more difficulties to distinguish between patients with and without need for help and support for oral hygiene by nursing staff or nursing relatives. Although the TTMC&NG is not generally used in every geriatric hospital, it has the potential to support dental and non-dental professionals in this decision process, since performance and interpretation of the test does not require dental knowledge.

Nursing staff or nursing relatives play a crucial role to safeguard oral hygiene and oral health in geriatric patients. Therefore, it is mandatory to raise attention, responsibility, knowledge, and motivation towards oral problems in caregivers and to empower them to face this task.

Several limitations of this study are to be considered. First, the study was conducted in the limited setting of one geriatric clinic. Therefore, generalization and transferability to other settings should be analyzed in further investigations. Second, due to limited personal resources, the same dentist conducted the TTMC&NG and the oral examinations, which could be considered a risk for observation bias. In further studies, it would be preferable to have two blinded investigators, one running the TTMC&NG and another conducting the oral examinations. Third, the TTMC&NG was performed once, failing to detect performance variations due to changes in the general health status and environment. When predicting the ability to perform oral hygiene, it should be considered that passing the test does not necessarily imply good quality oral hygiene, especially in patients with acute medical conditions, inadequate caregiver support, or low oral health literacy. Although direct observation of performing the TTMC&NG may clarify discrepancies between observations and self- and proxy perceptions of the patients’ abilities, the TTMC&NG did not detect the everyday performance in the home environment.

## Conclusion

The TTMC&NG served as a suitable predictor for the ability of geriatric inpatients to perform autonomously effective tooth brushing and denture cleaning. It might help geriatric medical staff to identify geriatric inpatients unable to perform effective oral hygiene by themselves.
